# 

**Published:** 1848-07

**Authors:** 


### THE MISSISSIPPI VALLEY ASSOCIATION OF DENTAL SURGEONS AND THE REGISTER.

We publish, in this No. of the Register, the Constitution of the Mississippi Valley Association of Dental Surgeons, and also append the list of officers for the present year. We would draw attention to the eighth article as amended, and hope to see every member of the Society present and comply with its requisitions. We should be glad to see a sufficient amout of funds in the Treasury to justify a handsome award for the best specimen of mineral teeth; and as perfection is not yet attained in their manufacture, it might be the means of stimulating to further improvement.

As the Register will contain the only published notice of our approaching meeting, it ts hoped that each member of the Society will bear the time in mind, and also take some pains to let their friends know, who may wish to become members. We shall send a few numbers of our present issue to non-subscribers, who, we know, wish to know the time of our mecting, and we hope it may induce them to send for the back numbers of the first Vol· of the Register.

The present No. completes the 1st vol., making a work of some two hundred and ten to twenty pages, most of which is original and practical matter, the object having been to furnish to the readers of the Register such information as might be of service in their practice.—CIN. ED.

---

### "WAXED CLOTH CONES."

### OHIO COLLEGE OF DENTAL SURGERY.

The Annual Announcement of this Institution will be out in a few days. Arrangements are such as to place the school on a secure and permanent footing, and at the same time give the dental student advantages for the acquisition of theoretical and practical knowledge, no where to be found out of a Dental College.

Every member of the Profession should feel an tnterest in sustaining such Institntions. There are now one in the East and one in the West, and if every dentist would exert his proper influence, both would be well sustained.

The idea that those alone engaged in teaching are interested in such enterprises, is very erroneous. True, they individually make greater sacrifices to elevate the standard of our Profession; yet they are not really more interested in its advancement than *should* be every *member* of the Profession. Our practice is all alike more or less affected by the pretensions and abuses of the empiric, and we are satisfied that the only true way to overcome this evil is to educate the Profession. CIN. ED.

### TO SUBSCRIBERS.

The present No. completes the first vol. of the Register; and we wish it borne in mind, that payment for subscription is expected always in *advance.* Those, therefore, who wish to secure the Nos. of the second vol. will remit two dollars, the amount of subscrirtion, before the next No. is issued.—Cin. Ed.

---

### DENTAL MIRROR AND ANNUAL VISITER.

We received, some two years since, from our friend Dr. Bridges, a copy of the above. Will the Dr. renew thus again our acquaintance. We send him some of our gossip in exchange for his.—Cin. Ed.

---

### DENTAL ADVERTISEMENTS.

We hope that members, coming in to the meeting of the Society, will bring in anything in this line that is rare or extraordinary that they may meet with, as the Society intends preserving a budget of specimens in their cabinet of curiosities, and some of which may perhaps be published in the Register *gratus.*—E. T.

### OHIO COLLEGE OF DENTAL SURGERY.

The regular course of Lectures in this Institution commences on the first Monday in November, 1848.

The Faculty is now complete by the appointment of John T. Shotwell, M. D. to the chair of Anatomy, and Physiology, and George Mendenhall, M. D. to the chair of Pathology and Therapeutics. Charles H. Raymond will deliver a course of Lectures on Chemistry, with special reference to Dentistry.

It is needless to speak particularly of these gentlemen who are known so well to the Medical Profession as able and efficient lecturers in their respective departments.

The faculty now stands as follows. The price of each ticket is annexed.

| | | |
|---|---|---|
| JAMES TAYLOR, M. D. D. D. S. Professor of Theory and Practice of Dental Surgery | Ticket | $25,00 |
| JOHN T. SHOTWELL, M. D. Professor of Anatomy and Physiology | " | 15,00 |
| GEORGE MENDENHALL, M. D. Pıofessor of Pathology and Therapeutics | " | 25,00 |
| CHARLES H. RAYMOND, M. D. Lecturer on Chemistry | " | 15,00 |
| A. M. LESLIE, D. D. S. Demonstrator of Mechanical Dentistry | " | 15,00 |
| Matriculation Fee | | 5,00 |
| Full course | | $100,00 |
| Diploma Fee | | 25,00 |
| The Demonstrator's ticket of Anatomy, (optional,) | | 10,00 |

### TERMS OF ADMISSION AND GRADUATION.

Candidates for graduation will be required to have attended two full courses of Lectures, the latter of which shall have been in this institution.

A full course in a respectable Medical or Dental College will be acknowledged as equivalent to a course in this Institution.

The candidate must be twenty-one years of age, of good moral character, and have made suitable attainments in the profession.

A reputable practitioner in Dentistry, who has been four years or more in practice, upon producing satisfactory testimonials of the same, shall be entitled to an examination for a degree, after attending one full course in this Institution.

Each candidate will be required to present and defend before the Faculty, a written Thesis on some subject relating to the science of Dentistry. He will also be required to deposit in the Cabinet of the College a full set of teeth of his own workmanship, and be subject to a critical examination upon the various branches of science taught in the College.

JAMES TAYLOR, *Dean.*

### CONTENTS OF VOLUME 1.

Address opening by, E. Taylor, D. D. S. 5
" on Tobacco, by A. Berry, D. D. S. 12
" Introductory, by E. Slack, M. D. 115
" Valedictory, by J. Taylor, M. D. D. D. S 173
Advertisements, Quack 216
Allen, J. D. D. S. on the contour of the face, 24, 103
" on destroying nerves, 25
" on Professional jealousy, 164
" Dentists in Cincinnati, 211
Amalgum Galvanic, action of, E. Slack, M. D. 18
Ambler's Journal of Dent. operations, 170
American Society of D. S. review of proceedings, 93
" " " comments on proceedings of, 134
" " " and Dental Register, 189
Anecdote by H. Crane, D. D. S. 46
Annealing gold foil, St. Louis Ed. 111
Anæsthetic agent, Chloroform, 85
Anæstheticised, a medical staff, 152
Association, Miss. Val., of Dental Surgeons 1
" " " Constitution of 179
" " " notice of 225
Berry, A. D. D. S. Address on Tobacco 12
" " letter from 153
" " Physicians acting Dentist 204
" " Third Dentition in early life 205
Brown, B. B. M. D. D. D. S. letter from 57
" " Editorials 109, 110, 111
" " Comments on the action of the A. S. of D. S. 194
Brevities by E. Taylor 106, 206, 207, 216
Calomel, effects of on the teeth, J. Taylor, M. D. D. D. S, 194
Capping the nerve, by B. T. Whitney, M. D 156
Cast boxes for soldering in 206
Carter, Dr. R. C. Tooth-ache, recipe for 48
Chloroform 85
" notice of, 107
" death from 148
" and Ether, notice of 169
Children's teeth, filling of 213
Cincinnati, Dentists in, 211

Cleaning files, ........ 110
College, Ohio of Dental Surgeons, ........ 53
College, Ohio, of D. S. ........ 168, 226
Comments on the Pro. of A. S. D. S. ........ 134
Cones, Waxed Cloth ........ 225
Constitution of the M. V. A. of D. S. ........ 179
Contour of the Face ........ 24, 103
Crane, H., D. D. S., Letter from ........ 45
Do do an Anecdote ........ 46
Do do Facts for the People ........ 82, 132
Cutlery ........ 110
Cutting off the Crowns previous to inserting on Pivot ........ 107
Death from inhalation of Chloroform ........ 148
Decay of Teeth, Popular Prejudices ........ 53
Dentists in Cincinnati ........ 211
Dental Surgeons, Miss. Valley Ass. ........ 1
Do do do Constitution of ........ 179
Do do do Notice of ........ 225
Do Am. Society of, Review of Proceedings ........ 93
Do do Comments on ........ 134
Do do and Dental Register ........ 189
Dental Surgery, Ohio College of ........ 53, 168, 226
Do Harris' Principles and Practice ........ 160
Dentition, Case of early Third ........ 205
Do do Third ........ 39
Dentistry, Harris' Work on ........ 111
Destroying Nerves ........ 25
Do do and Capping ........ 156
Editorial ........ 49, 50, 54, 107—112, 166—170, 219—227
Excessive Hæmorrhage from ext. ........ 158
Extraction and Structure of Teeth ........ 199
Filling Teeth ........ 27, 73, 182
Furnace ........ 154
Gutta Percha ........ 215
Galvanic Action ........ 18
Gen. Tom Thumb ........ 166
Gums Diseased ........ 30
Hill's Soft Filling ........ 226
Holders, Teeth ........ 106
Hoy, Dr., on the furnace ........ 154
Hullihen, Dr., Letter from ........ 47
Hæmorrhage from Ext. Tooth ........ 158
Ide, W. E., D. D. S., on whole sets of Teeth ........ 78, 208
Do do Notice of Removal ........ 170
Improved Mouth Plates ........ 223
Lathe, by A. M. Leslie ........ 63
Letheon ........ 40
Do Use of ........ 41

Letheon, Rules for Exhibition .......... 44
Letter from H. Crane, D. D. S. .......... 45
Do S. P. Hullihen .......... 47
Do St. Louis Editor .......... 57
Do H. R. Smith, D. D. S. .......... 105
Do A. Berry, D. D. S. .......... 153
Do Dr. J. Y. Shirley .......... 186
Leslie on the Lathe .......... 63
Locke, Erie, D. D. S., on the Structure of the Teeth .......... 199
Miss. Val. Ass. of Dental Surgeons .......... 1, 109, 179
Nerves, Destroying of Human Teeth .......... 25
Nerve, Destroying and Capping .......... 156
New Method of Operating .......... 112
Novel Modes of Extracting Teeth .......... 212
Ohio College of Dental Surgery .......... 53, 168, 226
One of the Objects of the Register .......... 216
Phrenology .......... 207
Physicians acting Dentists .......... 204
Popular Prejudices on Decay of Teeth .......... 53
Professional Gossip .......... 221
Professors Cook and Rodgers .......... 169
Quack Advertisements .......... 216
Review of the Proceedings of A. S. of D. S. .......... 93
Register, Dental, of the West .......... 51, 52, 108
Repairing Instruments .......... 109
Rogers, M., D. D. S., on Nerve Killing .......... 34
Ross, W. B., on Vitality of the Teeth .......... 70
Sanford, Dr. J., on Third Dentition .......... 39
Slack, E., M. D., on Galvanic Action .......... 18
Do do Introductory Address .......... 115
Smith, R. H., D. D. S., on Diseases of the Gums .......... 30
Do do do Letter from .......... 105
Shirley, Dr. J. Y., Letter from .......... 186
Staple Rivet Teeth .......... 48
Structure of the Teeth, &c. .......... 199
Subscribers, to .......... 170, 227
Taylor, Jos., D. D. S., on the Use of the Letheon .......... 41
Taylor, James, M. D., D. D. S., on Filing Teeth .......... 142
Do do do Valedictory Address .......... 173
Do do do on the Effects of Calomel .......... 194
Taylor, E., D. D. S., Review of the Proce. of A. S. of D. S. 93
Do do on Filling Teeth .......... 27, 73, 182
Do do Brevities .......... 106, 206, 207, 216
Valedictory Address, by James Taylor, M. D., D. D. S. .......... 173
Vitality of the Teeth .......... 70
Wax, Adhesive .......... 107
Waxed Cloth Cones .......... 225
Wharton, Duct of, Removal of a Foreign Body from .......... 218
Whole Sets of Teeth, by W. E. Ide, D. D. S. .......... 78. 108

